# A Tick of the Clock: Finding the Sweet Spot in Tilt Table Test. The Effectiveness of Short‐Duration Head‐Up Tilt Test as a Diagnostic Tool in Suspected Vasovagal Patients: A Retrospective Observational Study in a Tertiary Syncope Unit

**DOI:** 10.1002/joa3.70190

**Published:** 2025-09-25

**Authors:** Parvin Kalhor, Parichehr Ghahari, Nader Asgari, Arash Jalali, Saeed Sadeghian

**Affiliations:** ^1^ Cardiovascular Diseases Research Institute, Tehran Heart Center Tehran University of Medical Sciences Tehran Iran; ^2^ Doctor of Medicine Tehran University of Medical Sciences Tehran Iran

**Keywords:** head‐up tilt test, syncope, tilt‐table test, vasovagal syncope

## Abstract

**Aims:**

The head‐up tilt test (HUTT) has been markedly changed over the years, especially in the specified time for the passive and active phases. However, a consensus‐based protocol has yet to be established.

**Methods:**

Seven hundred twenty‐four patients suspected of vasovagal syncope who underwent HUTT through one of the protocols of 15to 20‐min testing for each active/passive phase (the whole test duration was 30 or 40 min, respectively) were evaluated. Then, the positive responses were recorded.

**Results:**

470 (64.9%) and 254 (35.1%) patients in the 15‐ and 20‐min groups, respectively. Overall, 238 patients (50.6%) in the 15‐min group and 140 patients (55.1%) in the 20‐min group had positive responses (*p* = 0.25). There was no significant difference in the number of positive responses between the 15‐ and 20‐min groups in any of the passive (*p* = 0.53) and active (*p* = 0.3) phases.

**Conclusion:**

The 15‐min HUTT protocol has similar results to the 20‐min protocol. Saving 10 min for each test has several potential benefits, such as increasing patient acceptance, decreasing patient discomfort, and enabling the conduct of more tests in a day in a syncope unit.

## Graphical abstract

In patients with suspected vasovagal syncope, a 15‐min head‐up tilt test protocol yielded similar diagnostic results to the conventional 20‐min protocol. This shorter approach may improve patient comfort and syncope unit efficiency without compromising diagnostic accuracy.

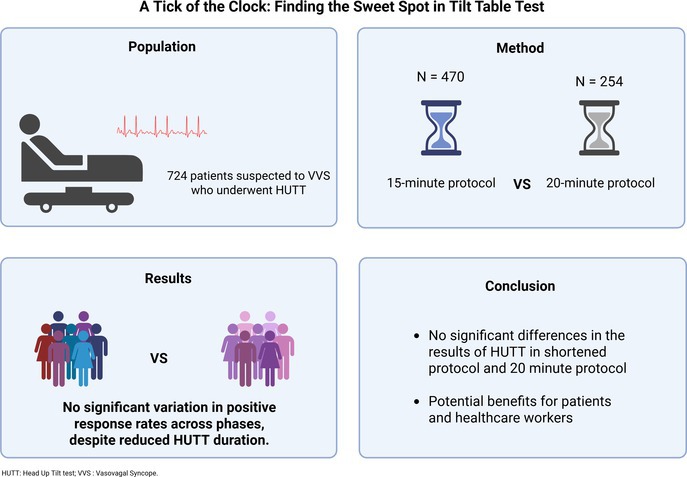

## 1 Introduction

Syncope, defined as an abrupt and transient loss of consciousness due to transient cerebral hypoperfusion with spontaneous recovery, is a common clinical problem with significant implications for patient quality of life and healthcare systems [1]. Vasovagal syncope (VVS), the most frequent cause of syncope, has a lifetime prevalence of up to 40% in adults and accounts for 1% to 1.5% of emergency department visits in the United States [2]. Although often considered benign, recurrent VVS can lead to significant morbidity, including injury, reduced quality of life, and anxiety related to unpredictable episodes [3, 4, 5].

The head‐up tilt test (HUTT) is a widely used diagnostic tool to evaluate suspected VVS, helping to reproduce syncope in a controlled setting by assessing autonomic function and cardiovascular response to orthostatic stress [6, 7]. The European Society of Cardiology (ESC) guidelines recommend HUTT as a valuable diagnostic tool, particularly when the diagnosis remains unclear after initial evaluation [1]. HUTT also aids in differentiating VVS from other causes of syncope, including orthostatic hypotension and cardiac arrhythmias, thereby guiding appropriate management strategies [1].

Despite its utility, the HUTT protocol has undergone significant modifications over the years, with ongoing debate regarding its optimal duration and structure [8]. Traditional protocols often employ a passive phase of 20 to 45 min, followed by a provocation phase using pharmacological agents such as nitroglycerin to increase diagnostic yield [9, 10]. However, prolonged test durations may lead to patient discomfort, increased resource utilization, and operational challenges within syncope units [11, 12]. Recent studies have suggested that shorter‐duration HUTT protocols may provide comparable diagnostic yield while enhancing patient tolerance and syncope unit efficiency [12].

Currently, there is no universally accepted consensus on the optimal HUTT duration, and the potential to reduce test time without compromising diagnostic accuracy remains an important area of investigation [1, 12]. In particular, it is unclear whether reducing the passive and active phases of the HUTT would impact the detection of VVS while preserving the safety and practicality of the procedure.

In this study, we aimed to evaluate the diagnostic yield of a shorter‐duration HUTT protocol in patients with suspected VVS by comparing the results of a 15‐min protocol with the conventional 20‐min protocol for both passive and active phases. We hypothesized that the shorter protocol would yield similar diagnostic results while offering potential benefits such as improved patient compliance, reduced discomfort, and increased operational efficiency within syncope units.

## 2 Methods

### 2.1 Study Population

We retrospectively evaluated patients with syncope who underwent HUTT over a four‐year period (2009–2013) at the Syncope Unit of Tehran Heart Center. According to the decision of the Tehran Heart Center Ethics Committee on October 29, 2017, this research project was ethically approved. Informed consent was obtained from all patients prior to documentation in the research project.

We included all patients with at least one episode of syncope or presyncope who had an unremarkable medical history, clinical examination, and electrocardiogram. Patients with a history of structural cardiac disease (e.g., valvular heart disease, cardiac tamponade, pulmonary hypertension, congenital heart anomalies) or primary arrhythmias, as well as those with neurological disorders, were excluded. Additionally, patients with specific causes of orthostatic hypotension leading to syncope were not included [1].

Suspected VVS was diagnosed based on clinical history obtained from patients, family members, and bystanders, along with physical examination and in accordance with the guidelines available at the time. In uncertain cases, VVS was diagnosed only after the exclusion of other potential causes of syncope. The diagnosis of VVS was not based on tilt testing; however, the HUTT was utilized to exclude alternative diagnoses such as orthostatic hypotension (defined as a decrease in blood pressure of ≥ 20/10 mmHg after 5 min of standing) and postural orthostatic tachycardia syndrome (defined as an increase in heart rate of ≥ 30 bpm after 5 min of standing).

Various HUTT protocols were employed depending on the patient's clinical presentation and the suspected etiology of syncope. At our center, two groups of physicians performed the HUTT. One group utilized a 15‐min protocol for both the passive and active phases, while the other group implemented a 20‐min duration for each phase. The specific protocol used varied based on the date of referral and physician preference.

### 2.2 HUTT Protocol

HUTT was performed in a dedicated, comfortable room with continuous monitoring of heart rate, blood pressure, and electrocardiography (CNS Systems, The Brain and Heart Company, Medizintechnik, Graz, Austria). Prior to testing, patients rested in the supine position for at least 5 min. During the passive phase, patients were tilted to a 70‐degree angle for 15 to 20 min according to their group. If syncope did not occur during the passive phase, 0.4 mg of sublingual nitroglycerin was administered to initiate the active phase. The test was considered positive based on the criteria outlined in the European Society of Cardiology guidelines for syncope [1]. In cases of syncope or significant symptoms, patients were immediately returned to the supine position for recovery. If none of the diagnostic criteria were met, the test was deemed negative, and patients were referred for further evaluation at the syncope clinic. During follow‐up, patients were interviewed regarding the clinical characteristics of their symptoms, including conditions before, during, and after the event, and appropriate treatment strategies were implemented.

A positive HUTT was defined according to the ESC 2018 guideline [1] as the reproduction of syncope or pre‐syncope associated with hypotension and/or bradycardia during the test, in the absence of other causes. Specifically:

Cardioinhibitory response: Defined as asystole > 3 s or severe bradycardia (HR < 40 bpm for > 10 s) associated with syncope/presyncope.

Vasodepressor response: Defined as a fall in systolic BP ≥ 50% from baseline or to < 60 mmHg, associated with symptoms, without severe bradycardia.

Mixed response: Presence of both hypotension and bradycardia meeting the above criteria with syncope/presyncope.

Borderline cases—such as asymptomatic hypotension or bradycardia, or symptoms without definitive hemodynamic changes—were classified as negative, and these patients were referred for further evaluation. Additionally, patients who were unable to complete the test due to severe nausea, vomiting, or agitation were also considered borderline and excluded from the study. Moreover, individuals who experienced loss of muscle tone without progressing to complete loss of consciousness were classified as borderline.

### 2.3 Statistical Analysis

Continuous variables were presented as mean ± standard deviation (SD) and were compared between 15‐ and 20‐min HUTT groups using a *T*‐test. Categorical variables were described as frequencies and absolute percentages and were compared between the groups by applying the chi‐squared test. Cumulative frequency and percentage of positive responses to HUTT over occurrence time were used. Data was analyzed by applying IBM SPSS Statistics for Windows, version 24.0 (Armonk, NY: IBM Corp.).

## 3 Results

In our study, 724 patients who underwent HUTT over a four‐year period were evaluated. At our center, HUTT was performed using two different protocols. One protocol consisted of 15 min for both the passive and active phases (total test duration of 30 min), while the other utilized 20 min for each phase (total test duration of 40 min). Accordingly, patients were classified into 15‐ and 20‐min protocol groups.

A total of 470 patients (64.9%) underwent the 15‐min protocol, while 254 patients (35.1%) underwent the 20‐min protocol. Table 1 presents the detailed baseline characteristics of both groups. As shown, there were no significant differences between the groups regarding gender, diabetes mellitus, and dyslipidemia. However, patients in the 20‐min group were older (49.4 ± 18.5 vs. 44.9 ± 18 years, *p* = 0.02) and had a higher prevalence of hypertension (29.3% vs. 16.8%, *p* = 0.004) compared to those in the 15‐min group.

**TABLE 1:** Baseline characteristics of patients undergoing the head‐up tilt test.

	15‐min for each phase (*n* = 470)	20‐min for each phase (*n* = 254)	*p*
Age	44.9 ± 18.0	49.4 ± 18.5	0.020
Gender (male)	123 (51.7%)	76 (54.3%)	0.624
Diabetes mellitus	21 (8.8%)	17 (12.1%)	0.300
Hypertension	40 (16.8%)	41 (29.3%)	0.004
Dyslipidemia	58 (24.4%)	43 (30.7%)	0.178

Overall, HUTT results were positive in 378 patients (52.2%) and negative in 346 patients (47.8%). Among the positive cases, 43 patients (11.4%) exhibited a positive response during the passive phase, while 335 patients (88.6%) exhibited a positive response during the active phase. Of the 378 patients with positive HUTT results, 179 (47.4%) were women, and 199 (52.6%) were men.

In the 15‐min group, HUTT was positive in 26 patients (5.5%) during the passive phase and in 212 patients (47.7%) during the active phase. In the 20‐min group, 17 patients (6.7%) had a positive response during the passive phase, while 123 patients (51.9%) had a positive response during the active phase. There was no significant difference in the rate of positive responses between the 15‐ and 20‐min groups during either the passive (*p* = 0.53) or active (*p* = 0.30) phases (Table 2). Overall, 238 patients (50.6%) in the 15‐min group and 140 patients (55.1%) in the 20‐min group had positive HUTT results, with no significant difference in the rates of positive responses between the groups (*p* = 0.25).

**TABLE 2:** Positive tilt test in the two 15‐ to 20‐min groups in active and passive phases.

	15‐min for each phase (*n* = 470)	20‐min for each phase (*n* = 254)	*p*
Passive phase	26/470 (5.5%)	17/254 (6.7%)	0.53
Active phase	212/444^a^ (47.7%)	123/237^a^ (51.9%)	0.3
Total positive response	238/470 (50.6%)	140/254 (55.1%)	0.25

^a^ Patients with a positive response during the passive phase were excluded from the active phase analysis.

**FIGURE 1:**
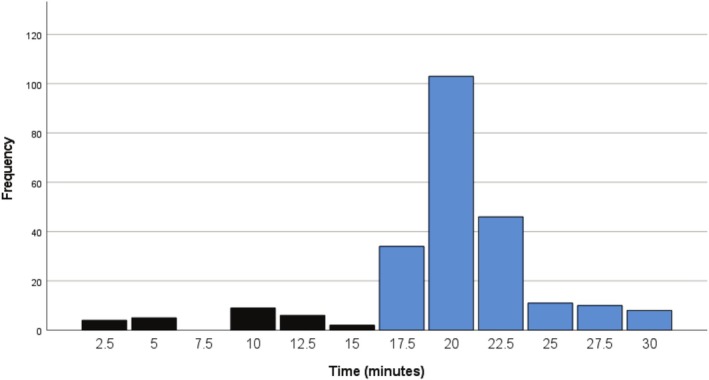
Number of patients with positive HUTT response (by 2.5 min time frame) during both passive (black colored) and active (blue colored) phases in the 15 min group.

**FIGURE 2:**
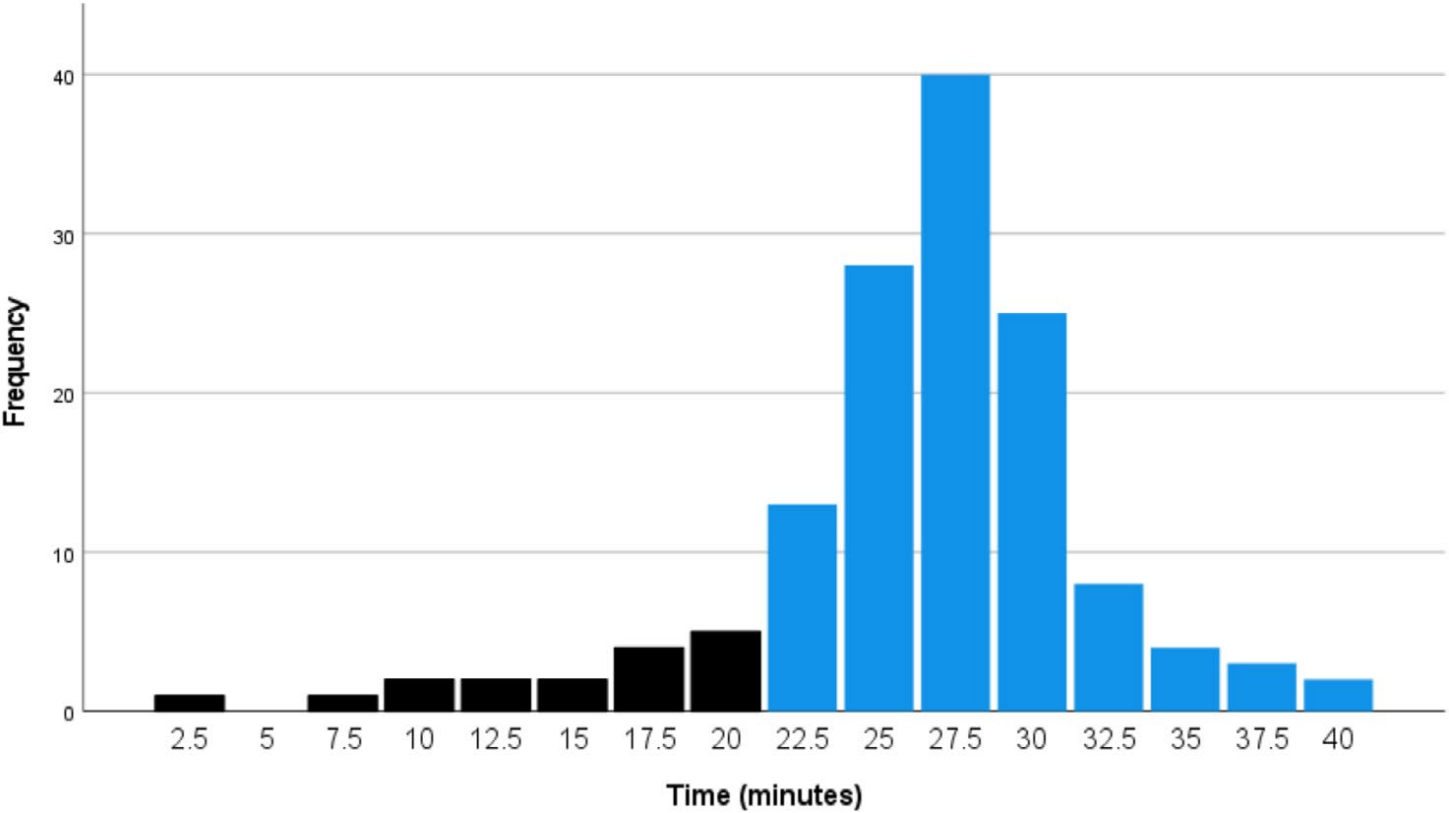
Number of patients with positive HUTT response (by 2.5 min time frame) during both passive (black colored) and active (blue colored) phases in the 20 min group.

Table 3 shows the frequency and cumulative frequency of positive HUTT responses at different time points across the two protocols. At the end of the passive phase, 10.9% of patients in the 15‐min group and 12.1% in the 20‐min group had positive responses. During the active phase, most patients in the 15‐min group exhibited a positive response within the first 5 min, resulting in 68.5% of patients demonstrating a positive response within the first 20 min of testing (from the start of the passive phase to the first 5 min of the active phase) (Figure 1). In the 20‐min group, 41.1% of patients exhibited a positive response within the first 25 min of testing (Figure 2).

**TABLE 3:** Frequency and cumulative frequency of positive responses to HUTT at different times in the two studied protocols.

Time (min)		15 min for each phase (*n* = 470) (Positive response: *n* = 238)					20 min for each phase (*n* = 254) (Positive response: *n* = 140)			
		Frequency	Percent	Cumulative frequency	Percent		Frequency	Percent	Cumulative frequency	Percent
2.5	Passive phase	4	1.7%	4	1.7%	Passive phase	1	0.7%	1	0.7%
5		5	2.1%	9	3.8%		0	0.0%	1	0.7%
7.5		0	0	9	3.8%		1	0.7%	2	1.4%
10		9	3.8%	18	7.6%		2	1.4%	4	2.9%
12.5		6	2.5%	24	10.1%		2	1.4%	6	4.3%
15		2	0.8%	26	10.9%		2	1.4%	8	5.7%
17.5	Active phase	34	14.3%	60	25.2%		4	2.9%	12	8.6%
20		103	43.3%	163	68.5%		5	3.5%	17	12.1%
22.5		46	19.3%	209	87.8%	Active phase	13	9.3%	30	21.4%
25		11	4.6%	220	92.4%		28	20%	58	41.4%
27.5		10	4.2%	230	96.6%		40	28.6%	98	70.0%
30		8	3.4%	238	100.0%		25	17.9%	123	87.9%
32.5							8	5.7%	131	93.6%
35							4	2.8%	135	96.4%
37.5							3	2.2%	138	98.6%
40							2	1.4%	140	100.0%

This trend continued throughout the test duration in both groups, with 92.4% of positive responses occurring within the first 25 min in the 15‐min group, compared to 87.9% of positive responses within the first 30 min in the 20‐min group. Additionally, as shown in Table 3, if the active phase duration in the 20‐min group had been reduced to 15 min, only five patients (3.6%) with positive responses would have been missed.

## 4 Discussion

The clinical evaluation of patients with suspected VVS suggests that HUTT is a valuable diagnostic tool. HUTT is considered safe, with no reported mortality or major complications [13]. However, the duration of the procedure presents practical challenges for patients, healthcare providers, and physicians. In this study, we evaluated patients with a clinical suspicion of VVS who were referred for HUTT. The tilt test was performed using two distinct protocols, each incorporating predefined durations for both the passive and active phases. The indication for HUTT and the criteria for a positive response were based on the guidelines of the ESC for the diagnosis and management of syncope [8, 14]. Patients were categorized into two groups according to the duration of the passive phase: a 15‐min group (*n* = 470, 64.9%) and a 20‐min group (*n* = 254, 35.1%). Among the 724 patients evaluated, 378 (52.2%) demonstrated a positive response to HUTT.

Among patients with a positive HUTT result, there was no statistically significant difference in response rates between the 15‐min group (238 patients, 50.6%) and the 20‐min group (140 patients, 55.1%) (*p* = 0.25).

Furthermore, analysis of the 20‐min group indicated that if the active phase had been terminated at 15 min, only 5 positive cases (3.6%) would have been missed. Notably, the shortened passive phase was associated with a marked increase in positive responses within the first 5 min of the 15‐min active phase. These findings suggest that reducing the total duration of HUTT by 10 min does not significantly impact diagnostic yield. The adoption of this abbreviated protocol may offer several potential advantages, including enhanced patient compliance, reduced anxiety, and improved efficiency, particularly important given the growing demand for tilt testing in syncope units. It is also noteworthy that current tilt testing protocols vary considerably in their recommended durations. The “Italian Protocol” recommends a 20‐min passive phase followed by a 15‐min active phase [15]. In contrast, the “Newcastle Protocol” prescribes a 40‐min passive phase and a 20‐min active phase [16]. Additionally, the 2018 ESC Guidelines for the diagnosis and management of syncope suggest a 20‐min passive phase and an active phase lasting 15 to 20 min [14].

Liu et al. [17] conducted a comprehensive study utilizing the HUTT to assess the prevalence and clinical characteristics of orthostatic hypotension. The study included 773 patients who underwent a 30‐min passive phase, followed by a 20‐min active phase in cases where syncope did not occur during the initial phase. To enhance diagnostic sensitivity, sublingual nitroglycerin was administered to induce vasodilation and evaluate the patients' circulatory response under pharmacological stress. The study reported a high overall positive response rate, with 55.2% of patients demonstrating features consistent with orthostatic hypotension. Notably, 10.8% of patients exhibited positive responses during the passive phase alone, suggesting an inherent predisposition to orthostatic intolerance. However, the majority of positive responses (89.2%) occurred following the administration of sublingual nitroglycerin, underscoring the enhanced sensitivity of pharmacologically augmented HUTT in detecting orthostatic hypotension.

During the active phase, the rate of positive responses increased rapidly following the administration of sublingual nitroglycerin, peaking at 10 min (35.7%) before subsequently declining. Notably, 96.1% of positive responses occurred within the first 15 min of the active phase. These findings are consistent with those of our study and suggest that a 15‐min active phase is sufficient for the effective detection of orthostatic hypotension.

In conclusion, the study by Liu et al. provides important insights into the prevalence, clinical features, and early identification of orthostatic hypotension via HUTT. The results support the use of a 15‐min active phase to optimize diagnostic efficiency and reinforce the value of HUTT as a practical tool in the clinical evaluation of syncope.

Khan et al. [12] conducted a study to assess the diagnostic performance of a short‐duration HUTT compared to the conventional protocol. The study included 100 patients, all of whom initially underwent a shortened HUTT consisting of two 15‐min phases. Patients with negative results were subsequently evaluated using the conventional HUTT protocol, which included a 30‐min passive phase followed by a 20‐min active phase.

The study demonstrated no statistically significant difference in diagnostic yield between the two protocols, with positive response rates of 53% for the short‐duration HUTT and 63% for the conventional protocol (*p* = 0.15). These findings align with our own results, supporting the feasibility of a shorter HUTT protocol as a practical alternative in selected clinical contexts.

Further research is warranted to assess the generalizability of these findings across diverse patient populations and to define the optimal role of short‐duration HUTT protocols in clinical practice.

Russo et al. [11] conducted a study to compare the diagnostic efficacy of two HUTT protocols in patients with suspected VVS. A total of 554 patients were randomly assigned to either a fast protocol group or a traditional protocol group. The fast protocol included a 5‐min pretest, a 10‐min passive phase, and a 10‐min active phase. In contrast, the traditional protocol consisted of a 5‐min pretest, followed by a 20‐min passive phase and a 15‐min active phase. The study found no significant difference in diagnostic event rates between the two groups, with 58.5% of patients in the fast group and 60.3% in the traditional group experiencing a syncopal event (*p* = 0.73). These findings suggest that shortening both the passive and active phases of the HUTT does not compromise its diagnostic yield in patients with suspected VVS.

Glocker et al. [13] conducted a study evaluating the temporal distribution of syncopal events during HUTT in patients with suspected vasovagal syncope. Their findings indicated a peak incidence of syncope between the 3rd and 5th min of the active phase, with events occurring within a range from the 1st to the 20th min. Notably, only 1.5% of patients experienced syncope after the 10th min.

These results are consistent with our findings, which demonstrated a 98.5% positive response rate following a 50% reduction in the active phase duration of HUTT. This evidence supports the premise that a shortened test duration may adequately capture the vast majority of diagnostic events in patients with vasovagal syncope.

Based on the cumulative evidence, a 30‐min HUTT protocol, comprising 15‐min passive and active phases, represents a reasonable balance between diagnostic accuracy and clinical practicality. Shortening the duration of both phases did not significantly affect diagnostic outcomes while providing several potential benefits, including improved patient compliance, reduced anxiety, and enhanced accessibility of the test. By minimizing the overall time commitment, the abbreviated protocol may facilitate wider utilization of HUTT in clinical practice, thereby promoting earlier diagnosis and more effective management of patients with orthostatic hypotension and suspected VVS.

It is important to recognize, however, that the HUTT supports the diagnosis of VVS; its prognostic value in predicting syncope recurrence remains uncertain. Previous studies have reported inconsistent findings regarding the utility of a positive HUTT result for risk stratification, with recurrence likely influenced more by clinical history and individual susceptibility than by the test outcome alone. Our study did not include a systematic follow‐up to evaluate recurrence rates, underscoring the need for further research to clarify the prognostic role of HUTT in guiding patient management.

## 5 Limitations

This study has several limitations. First, its retrospective design introduces inherent biases, and randomized allocation was not employed to ensure balanced group matching. Patient selection was determined by the attending physician, and the availability of HUTT influenced inclusion. Additionally, we did not systematically collect data on variables such as patient symptom characteristics, HUTT response types, blood pressure, and heart rate during testing. These limitations should be considered when interpreting the findings.

Given the retrospective nature of the study, the findings should be interpreted with caution. Further prospective multicenter research is needed to explore the generalizability of these findings across different patient populations and to establish the optimal HUTT protocol for the diagnosis of VVS. These limitations highlight the need for future research to address these issues through prospective designs and comprehensive data collection, including the systematic assessment of patient characteristics and physiological responses during the test.

## 6 Conclusion

Our analysis suggests that reducing the duration of both the passive and active phases of the HUTT does not significantly impact diagnostic outcomes in patients with suspected vasovagal syncope. This finding warrants further discussion and exploration within the medical community.

A 10‐min reduction in total test duration may offer several potential advantages, including:

- Improved patient compliance: Shorter tests may increase patient comfort and willingness to undergo evaluation.
- Reduced patient anxiety: A shorter testing duration may alleviate stress and improve tolerance, potentially enhancing the accuracy of test results.
- Increased efficiency: A shorter protocol enables evaluation of a higher volume of patients within the same timeframe, optimizing syncope unit operations.

While these benefits are promising, it is crucial to carefully consider the potential trade‐offs. Further research is needed to determine the optimal balance between test duration and diagnostic accuracy in different patient populations.

## Ethics Statement

The Ethics committee of the Tehran Heart Center approved this study.

## Conflicts of Interest

The authors declare no conflicts of interest.

## Data Availability

To protect study participant privacy, the whole data used and analyzed in this study is available from the corresponding author on reasonable request.
